# Efficacy of resveratrol in women with polycystic ovary syndrome: a systematic review and meta-analysis of randomized clinical trials

**DOI:** 10.11604/pamj.2023.44.134.32404

**Published:** 2023-03-16

**Authors:** Hammad Ali Fadlalmola, Amal Mohammed Elhusein, Khaled Mohammed Al-Sayaghi, Muayad Saud Albadrani, Duggahatti Veerabhadra Swamy, Daniel Mon Mamanao, Ehab Ibrahim El-Amin, Salma Elhadi Ibrahim, Siddiqa Mohammed Abbas

**Affiliations:** 1 Nursing College, Taibah University, Madinah, Saudi Arabia,; 2 College of Applied Medical Science, University of Bisha, Bisha, Saudi Arabia,; 3 College of Medicine, Taibah University, Madinah, Saudi Arabia,; 4 Jazan University, Faculty of Public Health and Tropical Medicine, Department of Epidemiology, Jazan, Saudi Arabia,; 5 Umm Alqura University, Faculty of Medicine, Algunfudah, Saudi Arabia

**Keywords:** Meta-analysis, polycystic ovary syndrome, resveratrol, systematic review

## Abstract

Polycystic ovarian syndrome (PCOS) is a metabolic and hormonal condition affecting women of a reproductive age. It causes an abnormal menstrual cycle, anovulation, infertility, acne, hirsutism, obesity, hyperlipidemia, and cardiovascular disorders. Because resveratrol decreases testosterone levels, it may be of value in treating PCOS. We aimed to evaluate the efficacy of resveratrol in treating women with PCOS. We searched for randomized clinical trials (RCTs) in PubMed, Cochrane CENTRAL, Scopus and Web of Science. With 95% confidence intervals, the data was retrieved and analyzed as a mean difference (MD) or a standardized mean difference (SMD). Four RCTs with 218 women were included in the analysis. Resveratrol significantly reduced testosterone (SMD = -0.40; 95% CI [-0.71, -0.10], P = 0.009), luteinizing hormone (LH) (SMD = -0.32; 95% CI [-0.62, 0.01], P = 0.04), and dehydroepiandrosterone sulfate (DHEAS) (MD = -0.85; 95% CI [-1.25, -0.45], P < 0.0001) compared with the placebo. Resveratrol is effective in treating women with PCOS due to reducing the levels of testosterone, LH, and DHEAS. In combination with other treatments, especially for hyperlipidemia, resveratrol is beneficial for women diagnosed with PCOS.

## Introduction

Polycystic ovary syndrome (PCOS) is a complex hormonal and metabolic disorder affecting women of reproductive age. The major features of PCOS are abnormal menstrual cycles, anovulation, infertility, acne, hirsutism, obesity, hyperlipidemia, and cardiovascular disorders (as part of the metabolic syndrome) [1-3]. Although the prevalence is 5%-7%, the etiology of the condition is not yet understood [4]. Hyperandrogenism is considered a critical aspect of this syndrome. Hyperplasia of the theca-interstitial (T-I) compartment in the ovary and the associated overexpression of many enzymes involved in steroidogenesis can result in an increased production of ovarian androgens [5-7]. PCOS may also be caused by increased levels of both fasting insulin as well as the relative ratio of the gonadotropin luteinizing hormone (LH) and follicle-stimulating hormone (FSH) [8]. Recently, evidence supported the theory that chronic low-grade inflammation plays an etiological role in the pathogenesis of PCOS. Many PCOS studies reported a strong association between inflammation and hyperandrogenism [9,10].

Efficient management for patients with PCOS is still controversial. Current treatments involve antiandrogens for androgen-related indications, including acne and hirsutism, clomiphene to induce ovulation, combined oral contraceptive pills (OCP) for both menstrual dysfunction and hyperandrogenism, and metformin as an insulin-sensitizing agent that may reduce the secretion of ovarian androgen [8]. Antiandrogens, such as flutamide and spironolactone, are well tolerated and have significant effects on hirsutism, but they may cause loss of libido, breast tenderness, irregular menstrual cycle, and the risk of changing a male fetus to a female. In pregnancy, there are many other adverse effects related to each drug in this category [11,12]. Clomiphene is considered one of the first options for managing anovulation; however, about 30% of the patients do not respond. In addition, it has particular constraints in patients with advanced age and a body mass index (BMI) >30 kg/m^2^ [8,13]. OCP induces a negative feedback of LH secretion and ovarian androgen synthesis may decrease. They could inhibit the peripheral conversion of testosterone to dihydrotestosterone (the active form). However, OCPs have several potential side effects, including insulin resistance, glucose tolerance, venous thrombosis, and vascular reactivity [14].

Alternative treatments are urgently required for PCOS patients. Resveratrol (trans-3,5,4’-trihydroxystilbene) is a natural polyphenol supplement found in grapes, nuts, and berries with recognized anti-inflammatory, antioxidant, anti-cancer, and cardioprotective activities [15]. In the last few years, the promising effects of resveratrol treatment for PCOS have been investigated. In vitro studies [16] indicated that exposing rat theca-interstitial cells (T-I) to resveratrol led to reduced androgen production by increasing executioner caspases, decreasing the number of viable cells, and inhibiting deoxyribonucleic acid (DNA) synthesis [17]. Recently, resveratrol administration decreased the level of testosterone in rats with dehydroepiandrosterone (DHEA)-induced PCOS. Banaszewska *et al*. performed the first clinical trial assessing the effect of resveratrol on PCOS and found a significant decline in ovarian and adrenal androgens [18]. The effect of resveratrol on ovarian steroidogenesis is selective, because it has no impact on the creation of progesterone by the theca cells [19].

Despite the beneficial results obtained by some clinical trials, the available data is still inconclusive and inadequate. Consequently, we performed systematic review and meta-analysis with all available RCTs to investigate whether resveratrol can improve menstrual irregularity, and symptoms related to hyperandrogenism and reduce the levels of androgens and insulin.

## Methods

**Search strategy and data collection:** we searched four databases: Cochrane CENTRAL, PubMed, Scopus, and Web of Science (WOS) for published clinical trials. The search included literature related to any current studies on the National Institutes of Health trials registry. The following search approach was used: (resveratrol or SRT501 or SRT-501 or resvida or stilbenetriol) and (“polycystic ovary syndrome” or “polycystic ovarian syndrome” or “Stein-Leventhal syndrome” or “sclerocystic ovary syndrome” or “sclerocystic ovarian degeneration” or “sclerocystic ovary” or “polycystic ovary disease” or “functional ovarian hyperandrogenism” or “ovarian hyperthecosis” or “Stein-Leventhal syndrome” or PCOS).

**Selection criteria:** we incorporated all RCTs for women with PCOS and evaluated the efficacy of the different doses of resveratrol to placebo. There was no age, gender, publishing date or location limits. Observational studies, animal studies, thesis reviews, non-English studies, non-accessible studies, and study abstracts were omitted.

**Study outcomes:** the efficacy of resveratrol in women with PCOS was measured through the following outcomes. Firstly, laboratory tests (hormones) including total testosterone (nmol/L), LH (mIU/mL), FSH (mIU/mL), TSH (mIU/mL), prolactin (ng/mL), and DHEAS (mol/L). Secondly, pregnancy occurrence rates including clinical and chemical pregnancy. Thirdly, lipid profiles including cholesterol (mg/dl), low-density lipoprotein-cholesterol (LDL-C) (mg/dl), high-density lipoprotein-cholesterol (HDL-C) (mg/dl), and triglycerides (mg/dL). Other parameters included an acne score, C-reactive protein (CRP), insulin (mIU/mL), and sex hormone binding globulin (SHBG) (nmol/L).

**Quality assessment:** the quality of the RCTs included were assessed by the Cochrane´s Risk of Bias Tool (Version 1, Chapter 8.5) [20]. According to the funnel-plot-based methods of Egger, due to the limited number of eligible studies, publication bias could not be evaluated [21].

**Statistical analysis:** the inverse-variance method was used to analyze the continuous outcomes as mean difference (MD) or the standardized mean difference (SMD) in cases of different units with a 95% confidence interval (CI). The Mantel-Haenszel method was used to analyze the dichotomous outcomes as relative risk (RR) with a 95% CI, and the fixed-effect model if the data were homogenous, if not, the random-effects model. We conducted the analysis with the Review Manager Software (version 5.3). In case of a missing standard deviation, we used Altman´s equation to calculate it from the 95% CI [22]. The equation is given as follows:


$$n=\frac{{\left(Z\alpha /2+Z\beta \right)}^{2}\left[p\left(1-p\right)\right]}{d^{2}}$$

Where n = sample size; Zα/2 = critical value of the standard normal distribution for a given level of significance; Zβ = critical value of the standard normal distribution for a given power (1-p); p = expected proportion of individuals with the outcome of interest in the population; and d = desired level of precision or effect size.

## Results

**Figure 1:**
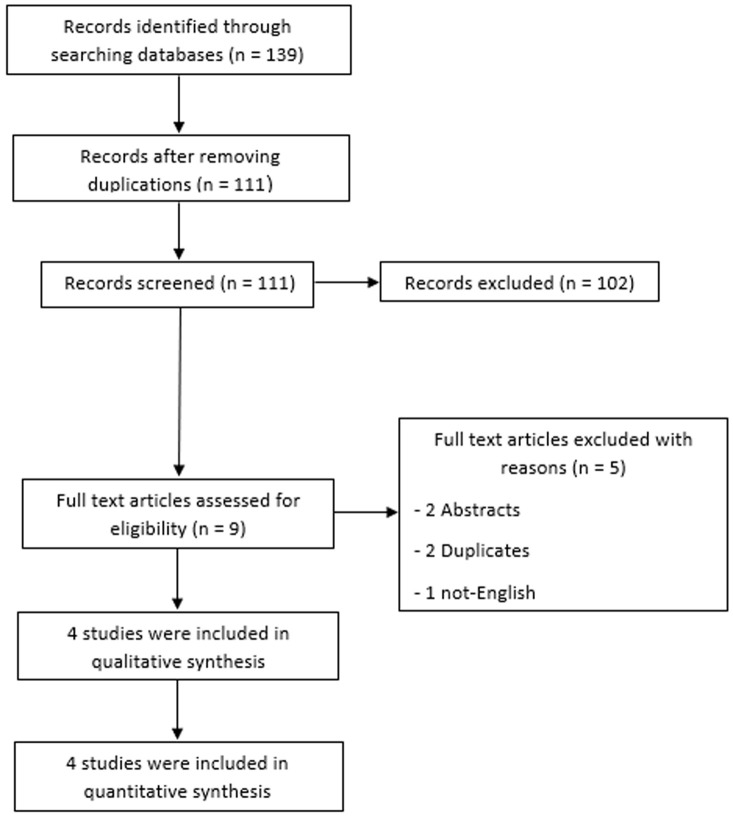
PRISMA flow chart which summarizes the literature search, and number of the obtained records

**Literature search results:** after removing the duplications, our search retrieved 111 unique citations; through title and abstract screening 102 were excluded and the 9 were eligible for full-text screening. Four RCT were eligible for inclusion [18,23-25]. Figure 1 displays the flow chart of the data collection and screening processes.

**Summary of the eligible RCTs:** the trials included compared different resveratrol doses (800, 1000, and 1500 mg daily) with the placebo. The total sample size was 218 women: 109 in each arm. All women were diagnosed with PCOS and received resveratrol or placebo orally, once or twice daily for at least 40 days. The women´s mean age ranged from 26.3 to 30.8 years. Table 1 and Table 2 display a summary of the trials as well as the baseline characteristics of the eligible participants.

**Table 1:** complete summary of the included studies and their findings

Study ID	Protocol number	Study arms, patients’ number	Resveratrol regimen	Country	Total sample	Follow-up duration	Inclusion criteria	Conclusion
Bahramrezaie 2019	IRCT2016030126860N1	Resveratrol, 31; placebo, 31	400 mg/twice daily, orally	Iran	62	40 days	Infertile PCOS patients, aged from 18 to 40 years, complying with the Rotterdam’s criteria. Except the PCOS, patients had no other endocrinopathies. All patients were candidates for intracytoplasmic sperm injection following 6 cycles of ovarian induction (Clomid and/or letrozole) with timed intercourse and 3 other cycles of IUI, which were unsuccessful in producing pregnancy (as a relative indication). There was no history of infertility among males.	Resveratrol might improve some PCOS outcomes, possibly through altering the serum levels of specific sex hormones and the HIF1 and VEGF genes expression in the angiogenesis pathway of the granulosa cells.
Banaszewska 2016	NCT01720459	Resveratrol, 17; placebo, 17	1500 mg/day, orally	USA	34	3 months	The sample fulfilled PCOS diagnostic criteria (Rotterdam consensus) and had two or more of the following: 1) hyperandrogenism (clinical or chemical); 2) oligomenorrhea or amenorrhea; and/or 3) polycystic ovaries as identified by the trans-vaginal ultrasound.	Resveratrol significantly lowered the ovarian and adrenal androgens, possibly related to an enhancement in insulin sensitivity and a reduction of the insulin serum level.
Brenjian 2018	IRCT2016041827453N1	Resveratrol, 22; placebo, 22	800 mg/day, orally	Iran	44	40 days	Women from 18 to 40 years, had PCOS (fulfilled Rotterdam criteria), with two or more of the following signs: (a) oligoovulation or anovulation, (b) biochemical and/or clinical signs of hyperandrogenism, and/or (c) polycystic ovaries as identified by the trans-vaginal ultrasound.	The resveratrol anti-inflammatory effects result the suppression of NF-κB and NF-κB regulated gene products. Resveratrol can also lessen ER stress in the granulosa cells (GCs) by changing the expression of the genes responsible for the unfolding protein response process. ER stress is a potential therapeutic target for patients with PCOS.
Mansour 2021	IRCT2017061917139N2	Resveratrol, 39; placebo, 39	1000 mg/day, orally	Iran	78	3 months	The sample fulfilled the PCOS diagnostic criteria (Rotterdam 2003 criteria), with two or more of the following three clinical findings: oligomenorrhea or amenorrhea, hyperandrogenism (clinical or chemical) and/or polycystic ovarian morphology as identified by ultrasound. Oligomenorrhoea was defined as a menstrual cycle >35 days. Amenorrhea was defined as no menses for 3-6 months or longer. Clinical hyperandrogenism was defined as the presence of acne, hirsutism and/or androgenic hair loss. Additional inclusion criteria were: age from 18 to 40 years and one month or more withdrawal of oral contraceptive pills or other steroid hormones, metformin and other drugs that could alter the metabolism (if taken before inclusion).	Resveratrol enhanced the menstrual cyclicity and hair loss, though changing the levels of androgens, lipids and insulin remained unchanged.

**Table 2:** baseline characteristics of enrolled patients in the included studies

Study ID	Arms, number of patients	Age	BMI (kg/m^2^)	Testosterone (ng/ml)	FSH (miu/ml)	LH (iu/ml)	Prolactin (ng/ml)	HS-CRP, (mg/l)	DHEAS (micro mol/l)	SHBG (nmol/l)	Acne score	HDL-c (mg/dl)	LDL-c (mg/dl)	Cholesterol (mg/dl)
Bahramrezaie 2019	Resveratrol, 19	29.30 (4.44)	25.92 (4.22)	1.02 (0.39)	3.89 (1.55)	10.65 (4.05)	11.61 (3.61)	-	-	-	-	-	-	-
	Placebo, 19	30.84 (3.30)	25.21 (4.28)	1.13 (0.49)	4.41 (1.49)	9.49 (4.62)	13.64 (5.99)	-	-	-	-	-	-	-
Banaszewska 2016	Resveratrol, 17	26.8 (4.5)	27.1 (6.18)	0.55 (0.165)	5.8 (1.24)	9.6 (5.77)	14.1 (7.42)	1.8 (1.65)	8.41 (2.85)	48.2 (20.6)	0.7 (0.82)	56 (13.61)	117.9 (32.16)	200.1 (33.81)
	Placebo, 17	26.8 (6.18)	27.6 (16)	4.7 (0.206)	5.1 (1.65)	8.4 (4.12)	15.3 (8.66)	2.7 (3.3)	8.09 (3.63)	43.5 (28.5)	0.4 (0.82)	51.3 (13.19)	117.9 (32.16)	169.1 (35.46)
Brenjian 2018	Resveratrol, 22	29.55 (3.28)	26.78 (3.81)	-	-	-	-	4.62 (3.38)	-	-	-	-	-	-
	Placebo, 22	30.35 (4)	30.35 (4)	-	-	-	-	3.66 (1.33)	-	-	-	-	-	-
Mansour 2021	Resveratrol, 39	26.33 (5.62)	-	-	6.26 (1.23)	8.49 (4.21)	13.5 (2.67)	0.95 (0.54)	166.89 ng/dl	50.64 (22.8)	1.42 (0.8)	36.4 (5.026)	91.38 (11.21)	169.62 (15.02)
	Placebo, 39	27.87 (6.24)	-	-	5.13 (1.085)	7.72 (3.69)	15.72 (3.11)	0.97 (0.55)	156.14 ng/dl	52.15 (23.15)	1.71 (0.762)	37.85 (5.23)	92.86 (10.85)	163.79 (15.06)

Data were represented as mean (SD); ID: identification; BMI: body mass index; FSH: follicle-stimulating hormone; LH: luteinizing hormone; HS-CRP: hormone sensitive-C-reactive protein; DHEAS: dehydroepiandrosterone sulfate; SHBG: sex hormone binding globulin; HDL-c: high-density lipoprotein-cholesterol; LDL-c: low-density lipoprotein-cholesterol

**Figure 2:**
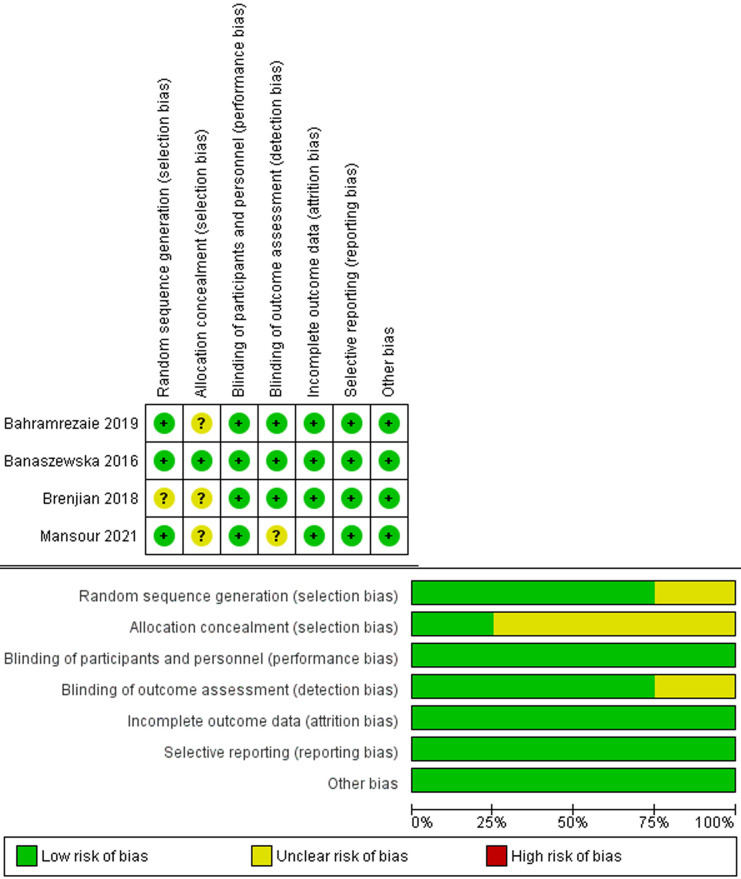
summary of risk of bias for each included study

**Quality assessment:** according to the Cochrane Risk of Bias tool, the eligible trials were high quality trials. All risk of bias were at low in terms of attrition, performance, reporting, and other sources of bias [18,23-25]. However, one trial [24] was categorized as an unclear risk of selection bias (randomization domain) and detection bias. Regarding selection bias (allocation domain), three trials [23-25] were low unclear and only one [18] was low risk. Figure 2 displays the risk of bias graph and summary.

### Outcomes

#### A. Laboratory tests (hormones)

**Figure 3:**
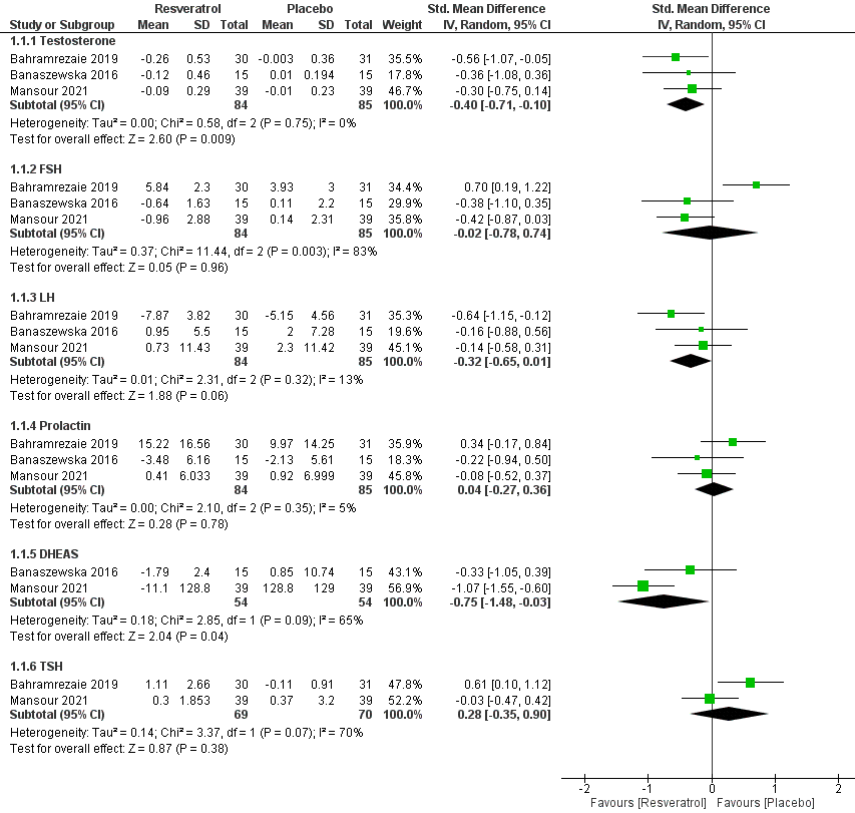
forest plot of laboratory tests (hormones) including total testosterone, FSH (mIU/mL), LH (mIU/mL), prolactin (ng/mL), TSH (mIU/mL), and DHEAS

**1. Total testosterone:** three studies [23-25] with analyzable data reported this outcome with a total of 169 patients; 84 in the resveratrol arm and 85 in the placebo arm. The pooled effect estimate indicated that resveratrol significantly reduced the level of testosterone, compared with the placebo (SMD = -0.40; 95% CI [-0.71, -0.10], P = 0.009) (Figure 3). The pooled results were homogenous (I^2^= 0%, P = 0.75).

**2. Follicle-stimulating hormone (FSH) (mIU/mL):** three studies [23-25] with analyzable data reported this outcome with a total of 169 patients; 84 in resveratrol arm and 85 in the placebo arm. The pooled effect estimate revealed no effect of resveratrol on FSH (SMD = -0.02; 95% CI [-0.78, 0.74], P = 0.96) (Figure 3). The pooled results were heterogenous (I^2^= 83%, P = 0.003), which was resolved by excluding Bahramrezaie 2019 [25] (I^2^= 0%, P = 0.93) and the results became significantly favoring resveratrol in term of reducing FSH (SMD = -0.41; 95% CI [-0.79, -0.02], P = 0.04).

**3. Luteinizing hormone LH (mIU/mL):** three studies [23-25] with analyzable data reported this outcome with a total of 169 patients; 84 in resveratrol arm and 85 in the placebo arm. Using the random effect model, the pooled effect estimate revealed that resveratrol significantly reduced testosterone compared to the placebo (SMD = -0.32; 95% CI [-0.65, 0.01], P = 0.06) (Figure 3). Using the fixed effect model, the pooled effect estimate indicated that resveratrol significantly lowered LH compared with the placebo (SMD = -0.32; 95% CI [-0.62, 0.01], P = 0.04). The pooled results were homogenous (I^2^= 0%, P = 0.75). The pooled results were homogenous (I^2^= 13%, P = 0.32).

**4. Prolactin (ng/mL):** three studies [23-25] with analyzable data reported this outcome with a total of 169 patients; 84 in the resveratrol arm and 85 in the placebo arm. The pooled effect estimate revealed no effect of resveratrol on prolactin (SMD = 0.04; 95% CI [-0.26, 0.35], P = 0.77) (Figure 3). The pooled results were homogenous (I^2^= 5%, P = 0.35).

**5. Thyroid stimulating hormone TSH (mIU/mL):** two studies [24,25] with analyzable data reported this outcome with a total of 139 patients; 69 in resveratrol arm and 70 in the placebo arm. The pooled effect estimate revealed that no effect of resveratrol on TSH (MD = 0.25; 95% CI [-0.09, 0.58], P = 0.15) (Figure 3). The pooled results were heterogenous (I^2^= 70%, P = 0.07), but the heterogeneity could not be resolved with only two trials included in the analysis of this outcome.

**6. Dehydroepiandrosterone sulfate (DHEAS):** two studies [18,24] with analyzable data reported this outcome with a total of 108 patients; 54 in each group. The pooled effect estimate revealed that resveratrol significantly reduced DHEAS compared with placebo (MD = -0.85; 95% CI [-1.25, -0.45], P < 0.0001) (Figure 3). The pooled results were heterogenous (I^2^= 65%, P = 0.09), but the heterogeneity could not be resolved with only two trials included in the analysis of this outcome.

#### B. Pregnancy occurrence

**Figure 4:**
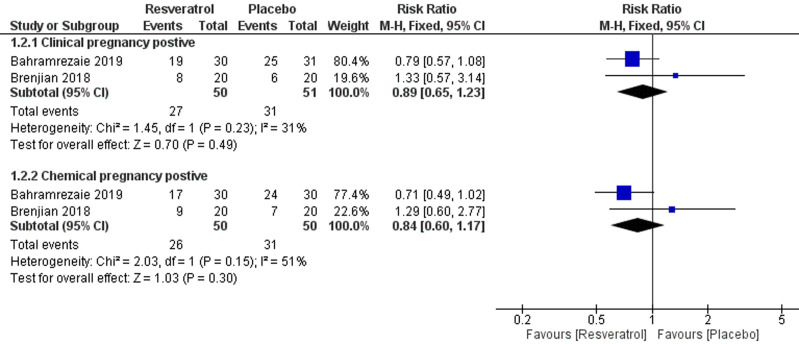
forest plot of pregnancy occurrence including positive clinical pregnancy and positive chemical pregnancy

**7. Positive clinical pregnancy:** two studies [23,25] with analyzable data reported this outcome with a total of 101 patients; 50 in the resveratrol arm and 51 in the placebo arm. The pooled relative risk (RR) indicated no effect of resveratrol regarding clinical pregnancy rates (RR = 0.89; 95% CI [0.65, 1.23], P = 0.49) (Figure 4). The pooled results were homogenous (I^2^= 31%, P = 0.23).

**8. Positive chemical pregnancy:** two studies [23,25] with analyzable data reported this outcome with a total of 100 patients; 50 in each arm. The pooled relative risk (RR) indicated that was no effect of resveratrol regarding clinical pregnancy rates (RR = 0.84; 95% CI [0.60, 1.17], P = 0.30) (Figure 4). The pooled results were homogenous (I^2^= 51%, P = 0.15).

#### C. Lipid profiles

**Figure 5:**
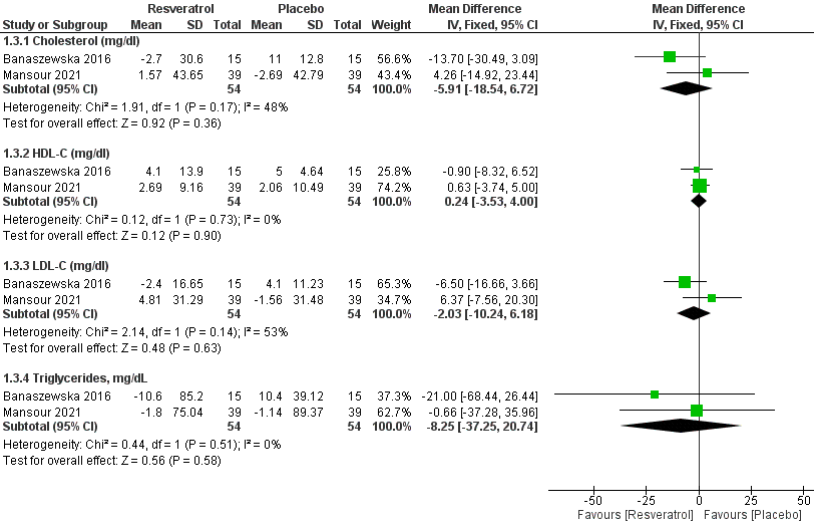
forest plot of lipid profiles including cholesterol (mg/dl), HDL-C (mg/dl), LDL-C (mg/dl), and triglycerides (mg/dl)

**9. Cholesterol (mg/dl):** two studies [18,24] with analyzable data reported this outcome with a total of 108 patients; 54 in each group. The pooled effect estimate revealed no effect of resveratrol on cholesterol (MD = -5.91; 95% CI [-18.54, 6.72], P = 0.36) (Figure 5). The pooled results were homogenous (I^2^= 48%, P = 0.17).

**10. High-density lipoprotein- cholesterol (HDL-C) (mg/dl):** two studies [18,24] with analyzable data reported this outcome with a total of 108 patients; 54 in each group. The pooled effect estimate revealed no effect of resveratrol on HDL (MD = 0.24; 95% CI [-3.53, 4.00], P = 0.90) (Figure 5). The pooled results were homogenous (I^2^= 0%, P = 0.73).

**11. Low-density lipoprotein-cholesterol (LDL-C) (mg/dl):** two studies [18,24] with analyzable data reported this outcome with a total of 108 patients; 54 in each group. The pooled effect estimate revealed no effect of resveratrol on LDL (MD = -2.03; 95% CI [-10.24, 6.18], P = 0.63) (Figure 5). The pooled results were homogenous (I^2^= 53%, P = 0.14).

**12. Triglycerides (mg/dl):** two studies [18,24] with analyzable data reported this outcome with a total of 108 patients; 54 in each group. The pooled effect estimate revealed no effect of resveratrol on triglycerides (MD = -8.25; 95% CI [-37.25, 20.74], P = 0.58) (Figure 5). The pooled results were homogenous (I^2^= 0%, P = 0.51).

#### D. Other parameters

**Figure 6:**
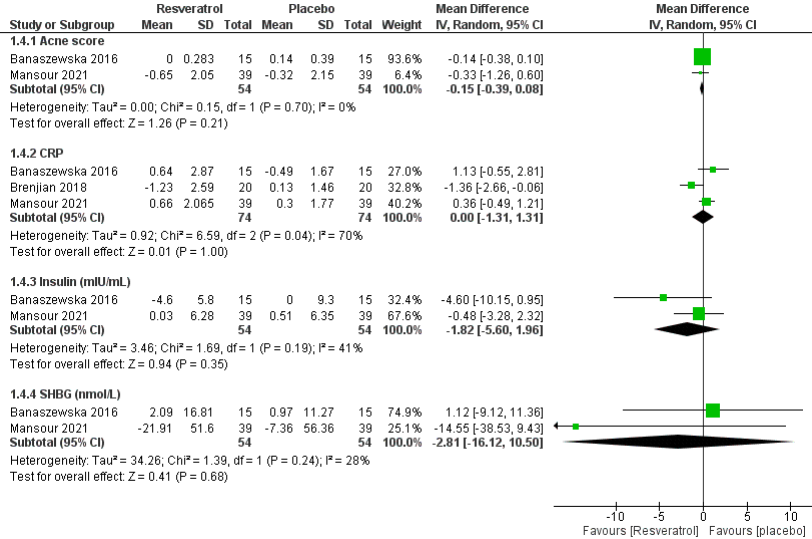
forest plot of other parameters including acne score, CRP, Insulin (mIU/mL), SHBG (nmol/L)

**13. Acne score:** two studies [18,24] with analyzable data reported this outcome with a total of 108 patients; 54 in each group. The pooled effect estimate revealed no effect of resveratrol on the acne score (MD = -0.15; 95% CI [-0.39, 0.08], P = 0.21) (Figure 6). The pooled results were homogenous (I^2^= 0%, P = 0.70).

**14. C-reactive protein (CRP):** three studies [18,23,24] with analyzable data reported this outcome with a total of 148 patients; 74 in each group. The pooled effect estimate revealed no effect of resveratrol on the CRP (MD = 0.00; 95% CI [-1.31, 1.31], P = 1.00) (Figure 6). The pooled results were heterogenous (I^2^= 70%, P = 0.04). Brenjian *et al*. 2018 was excluded to resolve the heterogeneity [23] (I^2^= 0%, P = 0.42) without affecting the significance (P = 0.18).

**15. Insulin (mIU/ml):** two studies [18,24] with analyzable data reported this outcome with a total of 108 patients; 54 in each group. The pooled effect estimate revealed no effect of resveratrol on insulin (MD = -1.82; 95% CI [-5.60, 1.96], P = 0.35) (Figure 6). The pooled results were homogenous (I^2^= 41%, P = 0.19).

**16. Sex hormone binding globulin (SHBG) (nmol/L):** two studies [18,24] with analyzable data reported this outcome with a total of 108 patients; 54 in each group. The pooled effect estimate revealed no effect of resveratrol on the SHBG (MD = -2.81; 95% CI [-16.12, 10.50], P = 0.68) (Figure 6). The pooled results were homogenous (I^2^= 28%, P = 0.24).

## Discussion

Based on the analysis, resveratrol had a significant effect on decreasing testosterone, LH, and DHEAS compared with placebo, indicating a positive effect on managing the hormonal imbalance responsible for most of the disease symptoms. However, there was a non-significant difference between the resveratrol and placebo for the FSH, prolactin, and TSH level. Resveratrol had no effect on the clinical or chemical pregnancy rates, the lipid profiles assessed by cholesterol, hormone-sensitive lipase (HSL), LDL, and triglyceride levels, or other parameters such as the acne score, CRP, insulin and SHBG.

The current treatment strategies used for managing women with PCOS include flutamide, spironolactone, metformin, clomiphene citrate, and combined OCP [8]. In patients with PCOS, anovulation is caused by high local androgen concentrations directly affecting the ovary [26,27]. Flutamide is a nonsteroidal antiandrogen of known efficacy in patients with hirsutism. It is not known yet whether it functions by only blocking the androgen receptors or through inhibiting androgen biosynthesis [27]. As flutamide therapy may reestablish ovulatory cycles and fertility, contraception may be required as reported by De Leo *et al*. [27]. Combined OCPs are the most frequently used treatment modality for PCOS as they suppress androgen production, and improve skin androgenic symptoms and menstrual dysfunction [28]. Vrbíková *et al*. reported prospective data about the effect of combined OC indicating a reduction in the risk of coronary artery disease and endometrial cancers in PCOS patients [28]. Clomiphene citrate (CC) is also one of the first therapeutic options for treating anovulation in PCOS patients due to its low cost, limited dose-dependent side effects, and ease of administration. Should the woman be CC resistant, metformin alone or with clomiphene is a feasible alternative [29]? According to Johnson 2014 [29], metformin is an appropriate first line of treatment in non-obese PCOS women with anovulatory infertility.

Regarding the current outcomes of the different hormone levels in PCOS, some hormone levels measured during the PCOS diagnosis include LH, FSH, total and free testosterone, prolactin, DHEAS, TSH, progesterone, androstenedione, and estrogen. Regarding the testosterone hormone level, the levels of both the total and free testosterone is often increased. A slight increase in testosterone can suppress normal menstruation and ovulation. In PCOS, the testosterone values will be ≤150 ng/dL (≤5.2 nmol/L) [30]. Our analysis revealed a significant effect of resveratrol, compared with placebo, in reducing the level of testosterone. The DHEAS values may be normal or slightly higher in PCOS [30]. The current analysis indicated a superiority of resveratrol over the placebo in decreasing the DHEAS level.

In terms of the LH and FSH hormones, which supports ovulation, women with PCOS typically have a LH level of 18 mlU/ml and a FSH level of 6 mlU/ml, which is within the normal range of 5-20 mlU/ml, and indicated as an elevated LH to FSH ratio or a ratio of 3: 1 [31]. Our analysis revealed that there was no significant difference between resveratrol and placebo in terms of FSH, with a marginal reducing effect on LH. Regarding the prolactin hormone, mild hyperprolactinemia occurs in 5% to 30% of PCOS patients [30,32]. Our results showed no superiority of resveratrol compared with placebo in decreasing the prolactin levels.

For the occurrence of pregnancy, clinical pregnancy decreases in PCOS due to hormonal disturbance. However, there was no difference between resveratrol and placebo. The lipid profile is frequently abnormal in PCOS patients [33], but the abnormalities are not consistent in all populations. According to Kiranmayee *et al*. [33] the majority (80%) of PCOS patients demonstrated abnormal lipid profiles, and in 70%, the abnormality included a low level of high-density lipoprotein (HDL) [33]. No superiority of resveratrol over placebo was observed in our analysis regarding a decrease in lipid profiles.

However, resveratrol produces significant results in some important outcomes. Bahramrezaie *et al*. [25] reported that resveratrol improves the PCOS symptoms, possibly through the expression of specific genes in the angiogenesis pathway of the granulosa cells or altering the serum levels of some sex hormones. Banaszewska *et al*. [18] concluded that the ovarian and adrenal androgens were significantly reduced by resveratrol, supporting an improvement in the condition. In addition, Mansour *et al*. [24] found that resveratrol improved the menstrual cyclicity and loss of hair. These findings could predict the potential effect of resveratrol, especially if combined with other lines of therapy to achieve the outcome, which cannot be obtained with resveratrol alone.

Regarding the strengths and limitations of our study, we conducted a comprehensive search to obtain a substantial level of evidence. The quality of the clinical trials we included complied with the currently accepted level of evidence. All the available outcomes in the final sample were reported. We performed a sensitivity analysis to detect the level of significance, and where heterogeneity occurred, it was resolved. However, some limitations should be considered. The publication bias could not be assessed due to the limited sample size. We included a small number of studies with a relatively small sample size. We recommend future clinical trials investigating the efficacy of resveratrol in combination with other lines of treatment, with a larger sample size and a longer follow-up duration.

## Conclusion

Resveratrol is a promising and effective drug in the treatment of women with PCOS due to its effect on the testosterone, LH, and DHEAS levels. Combinations with other treatment, especially for hyperlipidemia, are required to improve the outcome in women with PCOS.

### What is known about this topic

- *Polycystic ovary syndrome (PCOS) is a complex hormonal and metabolic disorder affecting women of reproductive age;*
- *The major features of PCOS are abnormal menstrual cycles, anovulation, infertility, acne, hirsutism, obesity, hyperlipidemia, and cardiovascular disorders*.

### What this study adds

- *Resveratrol is effective in treating women with PCOS due to reducing the levels of testosterone, LH, and DHEAS;*
- *In combination with other treatments, especially for hyperlipidemia, resveratrol is beneficial for women diagnosed with PCOS*.
